# The role of cancer-derived microRNAs in cancer immune escape

**DOI:** 10.1186/s13045-020-00848-8

**Published:** 2020-03-28

**Authors:** Ming Yi, Linping Xu, Ying Jiao, Suxia Luo, Anping Li, Kongming Wu

**Affiliations:** 1 Department of Oncology, Tongji Hospital of Tongji Medical College, Huazhong University of Science and Technology, Wuhan, 430030 China; 2 Department of Medical Oncology, The Affiliated Cancer Hospital of Zhengzhou University & Henan Cancer Hospital, Zhengzhou, 450008 China

**Keywords:** microRNA, Cancer immune surveillance, Immune escape, Immunotherapy, Tumor microenvironment, Exosome

## Abstract

During malignant transformation, accumulated somatic mutations endow cancer cells with increased invasiveness and immunogenicity. Under selective pressure, these highly immunogenic cancer cells develop multiple strategies to evade immune attack. It has been well established that cancer cells could downregulate the expression of major histocompatibility complex, acquire alterations in interferon pathway, and upregulate the activities of immune checkpoint pathways. Besides, cancer cells secret numerous cytokines, exosomes, and microvesicles to regulate the functions and abundances of components in the tumor microenvironment including immune effector cells and professional antigen presentation cells. As the vital determinant of post-transcriptional regulation, microRNAs (miRNAs) not only participate in cancer initiation and progression but also regulate anti-cancer immune response. For instance, some miRNAs affect cancer immune surveillance and immune escape by interfering the expression of immune attack-associated molecules. A growing body of evidence indicated that cancer-derived immune modulatory miRNAs might be promising targets to counteract cancer immune escape. In this review, we summarized the role of some miRNAs in cancer immune escape and discussed their potential clinical application as treatment targets.

## Background

Robust anti-cancer immune response consists of a series of stepwise immune events including the release of cancer-associated antigens, the processing and presentation of antigen presentation cells (APCs), the priming and activation of naïve T cells, the trafficking and migration of activated T cells, and the tumor-killing activity of effector cells [1, 2]. Actually, the anti-cancer immune response is a highly complex process which could be strengthened or weakened by multiple factors such as immune editing, transforming growth factor-β (TGF-β) signaling, and immune checkpoints [3–5]. The balance between immuno-stimulatory and -inhibitory factors is crucial to maintain the immune homeostasis of host and clear the cancer-derived materials [6]. However, some immunosuppressive factors could be hijacked by cancer cells to evade immune attack. With the advancement of cancer immunology, it has been realized that these immune rheostats might be ideal targets for cancer immunotherapy [7]. In the last decade, the application of immune checkpoint inhibitors greatly propelled the development of cancer therapeutics [8–10]. Blocking immune checkpoint-associated pathways effectively reactivates exhausted effector cells to eliminate cancer cells.

For some patients, the undermined cytotoxicity of effector cells is not the only rate-limiting step of eliminating cancer cells. In these patients, alterations in some upstream events in cancer-immunity cycle such as the recognition of cancer antigens, the functions of APCs, and the infiltration of T cells hamper cancer immune clearance as well [11, 12]. Additionally, more and more studies demonstrated that cancer-derived microRNAs (miRNAs) are closely implicated in the formation of the immunosuppressive tumor microenvironment, disabled effector cells, as well as downregulated cancer immunogenicity [13, 14].

miRNAs are a class of small, non-coding, single-strand RNAs which could silence target mRNAs by binding to corresponding 3′-untranslated region (3′-UTR) or open reading frame [15]. It is well documented that miRNAs participate in various physiological and pathological processes including immune defense, immune surveillance, immune homeostasis, as well as carcinogenesis [16–20]. Some specific miRNA patterns are highly correlated with cancer initiation, progression, and drug resistance [21, 22]. Notably, miRNAs could mediate the intercellular communication through being packed into exosomes or microvesicles [23]. As the vital post-transcriptional regulators, some immune modulatory miRNAs affect the expression of a broad range of immunity-associated genes in both cancer cells and tumor infiltrating lymphocytes (TILs) [24].

## Cancer immune escape

**Fig. 1:**
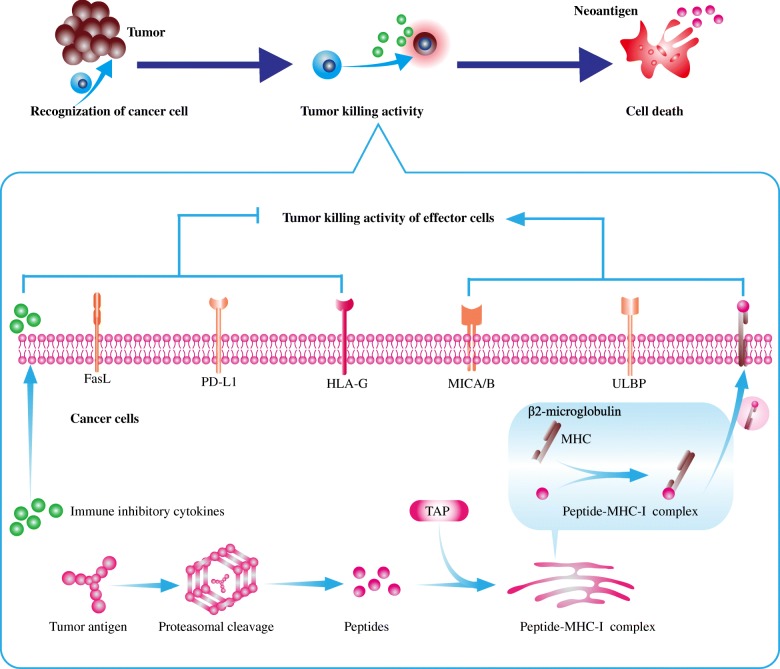
The mechanisms by which cancer cells escape from immune attack of immune effector cells. Firstly, tumor-derived cytokines especially TGF-β remarkably reshape the tumor immune microenvironment. This immunosuppressive cytokine repertoire inhibits the functions of multiple effector cells, induces the differentiation of regulatory cells, and impedes the infiltration of T cells. Secondly, overexpressed immune checkpoints or their ligands such as PD-L1 on cancer cells promote the formation of exhausted TILs. Thirdly, cancer cells tend to harbor alterations in antigen processing machinery, which result in the loss of tumor-associated antigens and neoantigens. Mutations in major histocompatibility complex class I (MHC-I), proteasome subunits latent membrane protein, as well as transporter associated with antigen processing reduce the presentation of recognizable targets on cancer cells. Fourthly, overexpressed HLA-G on cancer cells binds to the inhibitory receptors on effector cells such as CTLs and NKs, leading to the suppression of the cytotoxic activities of these effector cells. Lastly, cancer cells could escape immune attack by downregulating NKG2D ligands including MICA, MICB, and UL16-binding protein. *TAP* transporter associated with antigen processing, *MHC*-*I* major histocompatibility complex class I, *MICA*/*B* MHC-I chain-related molecules A/B, *IDO* indoleamine 2, 3-dioxygenase, *ULBP* UL16-binding protein

Although most patients have an intact immune system, some malignant cells could survive from immune attack and develop into clinically overt cancers [5]. Under the selective pressure of immune surveillance, cancer cells with high immunogenicity are preferentially eliminated by effector cells [25]. Eventually, cancer cells with weak immunogenicity escape immune clearance and become the predominant subpopulations [26]. The loss of immunoediting-mediated immunogenicity is a vital factor for cancer immune evasion. Moreover, other approaches could be utilized by cancer cells to produce immune evasion, such as inducing regulatory immune cells, acquiring disable antigen presentation machinery, upregulating immune checkpoints, and generating immunosuppressive microenvironment (Fig. 1) [27].

### Regulatory immune cells

Regulatory T cell (Treg) is an immunosuppressive class of CD4^+^ T cells [28]. In the tumor microenvironment, hyperactive Tregs could inhibit the tumor-killing activity of effector cells by secreting cytokines such as interleukin-10 (IL-10) and TGF-β [29]. Besides, Tregs promote cancer immune evasion by consuming IL-2 and upregulating the expression of multiple immune checkpoints including PD-L1, CTLA-4, T cell immunoglobulin and mucin domain-containing protein 3 (TIM-3), V-domain Ig suppressor of T cell activation (VISTA), as well as T-cell immunoreceptor with Ig and ITIM domains (TIGIT) [30–33]. It has been recognized that Tregs plays an indispensable role in ICI resistance at the present stage [34]. Similarly to Tregs, a subset of B cells are identified as immune inhibitory cells which are termed Bregs [35]. Bregs could inhibit inflammation response by increasing the generation of PD-L1 and cytokines such as IL-10 [36, 37].

Myeloid-derived suppressor cells (MDSCs) are a heterogeneous class of myeloid cell precursors which are halted at different stages of differentiation [38]. Abundant MDSCs in tumors induce cell cycle arrest of T cells via upregulating inducible nitric oxide synthase (iNOS) and arginase-1 (Arg1) [39]. Besides, MDSCs participate in oxidative stress and generate peroxynitrite which eventually blocks T cell activation [40]. Some MDSCs-derived materials such as IL-10, Arg1, and TGF-β could modulate the ratio of regulatory immune cells and effector cells [39]. Moreover, a specific phenotype of tumor-associated macrophages (TAMs), M2-type macrophages undermine anti-cancer immune response and promote immune evasion by anti-inflammation cytokines and immune checkpoint-associated pathways [41].

### Disable antigen processing and presentation of cancer cells

The cytotoxicity of cancer-specific effector cells is highly dependent on antigens expressed on cancer cells. Cancer cells tend to harbor alterations in antigen processing machinery (APM), which result in the loss of tumor-associated antigens (TAAs) and neoantigens [5]. Mutations in major histocompatibility complex class I (MHC-I), proteasome subunits latent membrane protein (LMP), as well as transporter associated with antigen processing (TAP) reduce the presentation of recognizable targets on cancer cells [42]. As a result, cancer cells exhibiting low immunogenicity are prone to survive from immune attack.

### Immune inhibitory cytokines and immune checkpoints

Tumor-derived cytokines especially TGF-β remarkably reshape the tumor immune microenvironment. This immunosuppressive cytokine repertoire inhibits the functions of multiple effector cells, induces the differentiation of regulatory cells, and impedes the infiltration of T cells [43, 44]. In addition, overexpressed immune checkpoints or their ligands such as PD-L1 on cancer cells promote the formation of exhausted TILs [45]. Moreover, some cancer cell-derived metabolites including indoleamine 2, 3-dioxygenase (IDO), arginase, and inhibitor of nuclear factor kappa-B kinase are greatly related to immune resistance in tumor as well [46–48].

## The role of miRNAs in cancer immune evasion

**Fig. 2:**
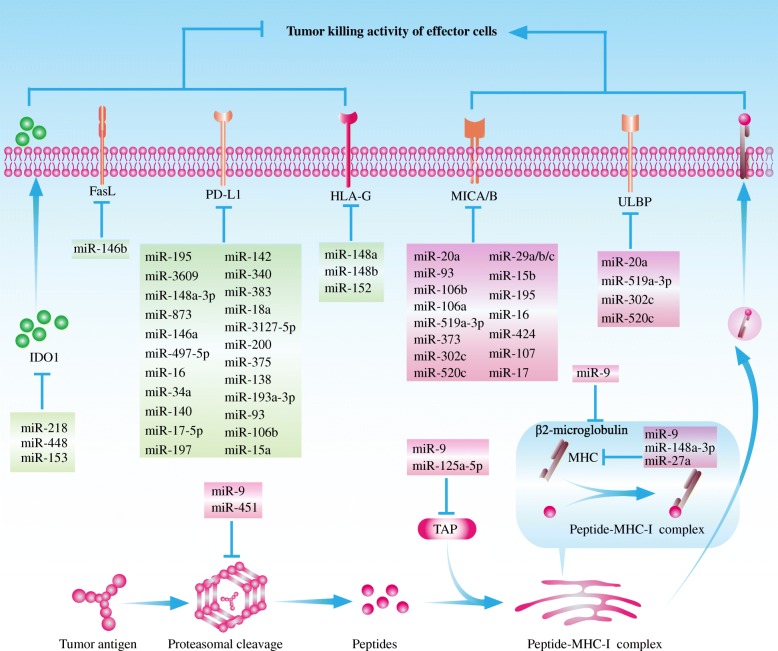
Cancer-derived miRNAs regulate immune evasion via modulating the expression profiles within cancer cells. Firstly, some immune modulatory miRNAs disturb antigen processing and presentation by targeting one or multiple components of APM (e.g., TAP1, LMP8, LMP9, and LMP10) and MHC-I molecules in cancer cells. Secondly, the loss of some HLA-G-targeting miRNAs is highly related with increased HLA-G expression, which is a well-accepted immune tolerant moelcule. Thirdly, cancer cells could escape immune attack by downregulating NKG2D ligands (MICA/B and ULBP2) in post-transcriptional level. Increased MICA/B- or ULBP2-targeting miRNAs protect cancer cells from immune clearence of NKs and CTLs. Fourthly, altered miRNA expressio pattern upregulates PD-L1 level in cancer cells. Lastly, dysregulated miRNA profiles change the metabolism of cancer cells. Increased IDO1 further hampered the normal immune survelliance. The miRNAs promoting anti-cancer immune response are exihibited in boxes with green background while the miRNAs inhbiting anti-cancer immune reponse are showed in boxes with red background. Upregualted immunostiumlatory miRNAs together with downregualted immunosuppressive miRNAs contribute to cancer immune evasion. *APM* antigen processing machinery, *TAP* transporter associated with antigen processing, *MHC*-*I* major histocompatibility complex class I, *LMP* proteasome subunits latent membrane protein, *MICA*/*B* MHC-I chain-related molecules A/B, *ULBP* UL16-binding protein, *CTL* cytotoxic T lymphocyte, *IDO* indoleamine 2, 3-dioxygenase

Apart from acting as tumor promoters or suppressors, it has been revealed that a growing body of miRNAs could regulate cancer immune surveillance and escape [49]. A panel of miRNAs protect cancer cells from immune clearance by decreasing the immunogenicity of cancer cells and downregulating the magnitude of anti-cancer immune response (Fig. 2). Simultaneously, another group of miRNAs strengthen anti-cancer immune clearance. These immune modulatory miRNAs are termed im-miRNAs [50]. Cancer cell-derived im-miRNAs not only target themselves but also broadly regulate various immune components including MDSCs, Tregs, DCs, NKs, as well as cytotoxic T lymphocytes (CTLs) via intercellular communication (e.g., exosomes and microvesicles) [24, 51, 52].

### The miRNAs regulating cancer antigen processing and presentation

Some im-miRNAs disturb antigen processing and presentation by targeting one or multiple components of APM and MHC-I molecules in cancer cells (Table 1). Specifically speaking, in nasopharyngeal cancer cells, the results of microarray profiles indicated that miR-9 could target several APM components including TAP1, LMP8 (also termed PSMB8), LMP9 (PSMB9), LMP10 (PSMB10), and β2-microglobulin [53]. Besides, miR-9 significantly downregulates MHC-I molecules including human leukocyte antigen-B (HLA-B), HLA-C, HLA-F, and HLA-H [53]. The overexpression of miR-9 in multiple cancers such as cervical cancer, non-small cell lung cancer (NSCLC), and glioma might contribute to enhanced immune tolerance in the tumor microenvironment [57–59]. Meanwhile, some endoplasmic reticulum stress-associated miRNAs such as miR-346 regulates immune response by directly targeting TAP1 or indirectly suppressing the expression of MHC-I molecules and interferon (IFN) signaling pathway [60].

**Table 1:** The miRNAs regulating cancer antigen processing and presentation

Targets of miRNAs	miRNAs	Cancer cell types	Ref.
TAP1	miR-9	Nasopharyngeal cancer	[53]
TAP2	miR-125a-5p	Esophageal adenocarcinoma	[54]
LMP8	miR-9	Nasopharyngeal cancer	[53]
	miR-451	Lung cancer	[55]
LMP9	miR-9	Nasopharyngeal cancer	[53]
LMP10	miR-9	Nasopharyngeal cancer	[53]
MHC-I	miR-9	Nasopharyngeal cancer	[53]
	miR-148a-3p	Esophageal adenocarcinoma	[54]
	miR-27a	Colorectal cancer	[56]
β2-microglobulin	miR-9	Nasopharyngeal cancer	[53]

*TAP* transporter associated with antigen processing, *LMP* proteasome subunits latent membrane protein, *MHC*-*I* major histocompatibility complex class I

Similarly, in esophageal adenocarcinoma cells, it has been verified that miR-125a-5p could bind to the 3′-UTR of TAP2 mRNA [54]. Also, it was detected that the 3′-UTR of HLA-A, HLA-B, and HLA-C mRNAs had the binding site for miR-148a-3p [54]. Meantime, the results of proteomic screening suggested that miR-27a promoted cancer progression by decreasing MHC-I expression on cell surface, inhibiting T cell infiltration and cytotoxic activities [56]. This miR-27a-induced MHC-I downregulation was dependent on calreticulin suppression [56].

### HLA-G-targeting miRNAs

As a non-classic MHC-I molecule, HLA-G was initially found to maintain fetal-maternal tolerance [61]. This immune inhibitory function of HLA-G could be hijacked by cancer cells to escape immune attack [62]. Overexpressed HLA-G on cancer cells binds to the inhibitory receptors on effector cells such as CTLs and NKs, leading to the suppression of the cytotoxic activities of these effector cells [62]. Actually, it has been found that HLA-G expression was aberrantly elevated in multiple cancers including melanoma, breast cancer, colorectal cancer, lung cancer, gastric cancer, hepatocellular carcinoma, and endometrial carcinoma [63, 64].

In cancer cells, the increased HLA-G expression is closely correlated with the loss of some HLA-G-targeting miRNAs. Specifically, several members of miR-148 family such as miR-148a, miR-148b, and miR-152 could downregulate HLA-G expression [65–67]. In breast cancer cells, it was observed that oncogenic estrogenic G-protein-coupled estrogen receptor-1 (GPER) signaling pathway decreased downstream miR-148a level, further contributing to cancer immune evasion [68]. Additionally, miR-133a was verified as a vital mediator in maintaining peripheral immune tolerance by targeting HLA-G [69].

### The MHC-I chain-related molecules A/B and miRNAs

The oncogenically transformed cells are susceptible to expressing a series of stress-induced ligands including MICA, MICB, and UL16-binding protein (ULBP) [70–72]. These ligands could be recognized by NKG2D on NKs and CTLs [70]. Intact NKG2D axis is an important signaling pathway to maintain cancer immune surveillance [73]. However, cancer cells could escape immune attack by downregulating NKG2D ligands in post-transcriptional level. So far, numerous miRNAs are portrayed as the modulators of NKG2D ligands (Table 2).

**Table 2:** The miRNA regulating NKG2D ligands

The targets of miRNAs	miRNAs	Cancer cell types	Ref.
MICA	miR-20a	Ovarian cancer	[74]
		Hepatocellular carcinoma	[75, 76]
		Breast cancer	[77]
		Colorectal cancer	[78]
		Gastric cancer	[79]
	miR-93	Hepatocellular carcinoma	[76, 80]
	miR-106b	Hepatocellular carcinoma	[76, 80]
	miR-106a	Hepatocellular carcinoma	[75]
	miR-519a-3p	Breast cancer	[81]
	miR-125b^a^	Multiple myeloma	[82]
	miR-373	Hepatocellular carcinoma	[75]
	miR-302c	Multiple cancers	[83]
	miR-520c	Multiple cancers	[83]
	miR-153^b^	Pancreatic cancer	[84]
MICB	miR-20a	Breast cancer	[77]
		Hepatocellular carcinoma	[75]
	miR-373	Hepatocellular carcinoma	[75]
	miR-29a/b/c	Hepatocellular carcinoma	[75]
	miR-15b	Hepatocellular carcinoma	[75]
	miR-195	Hepatocellular carcinoma	[75]
	miR-16	Hepatocellular carcinoma	[75]
	miR-424	Hepatocellular carcinoma	[75]
	miR-106a	Hepatocellular carcinoma	[75]
	miR-107	Hepatocellular carcinoma	[75]
	miR-17	Hepatocellular carcinoma	[75]
	miR-302c	Multiple cancers	[83]
	miR-520c	Multiple cancers	[83]
ULBP	miR-20a	Breast cancer	[77]
	miR-519a-3p	Breast cancer	[81]
	miR-302c	Multiple cancers	[83]
	miR-520c	Multiple cancers	[83]

*MICA*/*B* MHC-I chain-related molecules A/B (MICA/B), *ULBP* UL16-binding protein

^a^miR-125b upregulates MICA via targeting the transcriptional suppressor of MICA

^b^miR-153 promotes the formation of sMICA by targeting HIF1A pathway

Previous studies demonstrated that overexpressed miR-20a in colorectal cancer cells, breast cancer cells, and ovarian cancer cells decreases MICA level and sensitivity to immune effector cells [74, 77–79]. Besides, it was found that miR-519a-3p undermined the tumor-killing effect of NKs by decreasing MICA and ULBP2 on breast cancer cells [81]. In breast cancer patients, high miR-519a-3p expression could be deemed as a predictive biomarker for poor prognosis [81]. In addition, Abruzzese et al. noticed that bromodomain and extra-terminal (BET) inhibitor could remarkably increase MICA expression on multiple myeloma cells [82]. This BETi-induced MICA elevation was mediated by miR-125b, which targeted the transcription suppressor of MICA (IRF4) [82]. Moreover, Kishikawa et al. found that miR-93 and miR-106b targeted the 3′-UTR of MICA mRNA [80]. Genes coding miR-93 and miR-106b are both located in human chromosome 7q22.1 (termed miR25-93-106b cluster) [80]. Silencing miR25-93-106b cluster significantly increased MICA expression and decreased the susceptibility of hepatocellular carcinoma cells to NKs [80]. Similarly to the observations of Kishikawa et al., Wu et al. noticed that a panel of miRNAs downregulated MHC-I chain-related molecules A/B (MICA/B) in hepatocellular carcinoma cells including miR-373, miR-29b, miR-15b, miR-195, miR-16, miR-424, miR-29c, miR-106a, miR-107, miR-20a, miR-29a, as well as miR-17 [75]. Notably, 1, 25-(OH)2-D3 treatment could promote cancer immune surveillance by counteracting miR-302c/miR-520c-induced downregulation of MICA/B and ULBP2 downregulation [83].

On the contrary to MICA expressed on cancer cells (also known as membrane MICA), soluble MICA (sMICA) is an unfavorable factor for anti-cancer immunity [84]. In pancreatic cancer cells, hypoxia-associated pathways conspicuously downregulated membrane MICA while simultaneously increased sMICA expression [84]. This hypoxia-induced sMICA upregulation was attributed to the dysregulated balance between circ_0000977 and miR-153 [84].

### Immune checkpoint ligand-associated miRNAs

As a hallmark of cancer, upregulated immune checkpoint signal is determined by multiple factors, including previously existing inflammation and some oncogenic signaling pathways [85]. Increased immune checkpoint ligands especially PD-L1 is closely related with cancer-associated miRNA expression pattern. To be more specific, previous studies have indicated that the loss of miR-3609, miR-195-5p, miR-148a-3p, miR-873, miRNA-497-5p, miR-191-5p, miR-34a, and miR-138 closely correlated with the increased PD-L1 expression on numerous cancer cells [86–94]. In addition, Dong et al. found that decreased miR-140, miR-142, miR-340, and miR-383 enormously elevated PD-L1 expression on cervical cancer cells [95]. The results of a respective study demonstrated that in malignant pleural mesothelioma samples, the abundance of PD-L1 was negatively correlated with the levels of multiple cancer suppressive miRNAs including miR-15b, miR-16, miR-193a-3p, miR-195, and miR-200c [96]. Further investigation in cancer cell lines identified that miR-15b, miR-16, and miR-193a-3p could target PD-L1 mRNA [96]. Notably, some other long noncoding RNAs involved in the reduction of these PD-L1-targeting miRNAs [87, 94].

Contrarily, miR-18a promotes PD-L1 expression by targeting PTEN, WNK2, and SOX6 [95]. Then, PI3K-AKT, MEK-ERK, and Wnt/β-catenin pathways are activated and the transcription activity of PD-L1 is upregulated [95]. Similarly, Tang *et al.* observed miR-3127-5p induced PD-L1 expression by promoting STAT3 phosphorylation in NSCLC cells [97]. The miRNAs associated with PD-L1 expression on cancer cells were summarized in Table 3.

**Table 3:** The miRNAs regulating PD-L1 expression on cancer cells

miRNAs	Effects of miRNA on PD-L1 expression	Cancer cell types	Ref.
miR-195-5p	Downregulating	Pancreatic cancer	[87]
	Downregulating	Colon adenocarcinoma	[91]
	Downregulating	Prostate cancer	[98]
	Downregulating	DLBCL	[99]
miR-3609	Downregulating	Breast cancer	[86]
miR-148a-3p	Downregulating	Colorectal cancer	[88]
miR-873	Downregulating	Breast cancer	[89]
miR-146a	Upregulating	Melanoma	[100]
miR-497-5p	Downregulating	Clear cell renal cell carcinoma	[90]
miR-16	Downregulating	Prostate cancer	[98]
	Downregulating	MPM	[96]
miR-34a	Downregulating	B cell lymphomas	[92]
	Downregulating	Glioma	[93]
	Downregulating	AML	[101, 102]
miR-140	Downregulating	Cervical cancer	[95]
	Downregulating	NSCLC	[103]
miR-142	Downregulating	Cervical cancer	[95]
	Downregulating	NSCLC	[104]
	Downregulating	Pancreatic cancer	[105]
miR-340	Downregulating	Cervical cancer	[95]
miR-383	Downregulating	Cervical cancer	[95]
miR-18a	Upregulating	Cervical cancer	[95]
miR-3127-5p	Upregulating	NSCLC	[97]
miR-200 family	Downregulating	Lung cancer	[106]
	Downregulating	Hepatocellular carcinoma	[107]
	Downregulating	AML	[101]
miR-375	Downregulating	HNSCC	[108]
miR-138	Downregulating	Colorectal cancer	[94]
	Downregulating	Colorectal cancer	[109]
miR-15a	Downregulating	MPM	[96]
miR-193a-3p	Downregulating	MPM	[96]
miR-93	Downregulating	Pancreatic cancer	[110]
miR-106b	Downregulating	Pancreatic cancer	[110]
miR-17-5p	Downregulating	Melanoma	[111]
miR-197	Downregulating	NSCLC	[112]

*NSCLC* non-small cell lung cancer, *HNSCC* head and neck squamous cell carcinoma, *DLBCL* diffuse large B cell lymphoma, *MPM* malignant pleural mesothelioma, *AML* acute myeloid leukemia

### The miRNAs and tumor-mediated immune cell death

Fas-FasL pathway has a great impact on immune tolerance in the tumor microenvironment [113]. Increased FasL on cancer cells counterattacks immune cells, induces immune cell death, and eventually promotes cancer immune privilege [114]. In T cell large granular lymphocyte leukemia, it was found that STAT3-induced miR-146b loss led to increased FasL expression and potential neutropenia [115]. Besides, the miR-768-3p mimic treatment in NSCLC cell immensely increased FasL level but decreased Fas expression on cancer cells [116]. Moreover, Wu et al. verified that miR-21 could target FasL in breast cancer cells. In the co-culture experiment, ectopically expressed miR-21 in MCF7 cells could remarkably reduce the apoptosis rate of Jurkat T cells [117]. Actually, FasL has been accepted as the post-transcriptional regulatory target of miR-21 in numerous cancer cell types including esophageal carcinoma and pancreatic cancer [118–120]. Notably, in spite of inhibiting immune evasion, highly expressed miR-21 was connected with cancer development, treatment resistance, and poor prognosis [118, 119].

### Cancer cell metabolite-related miRNAs

Some cell metabolites such as tryptophan are essential to maintain the functions of TILs. IDO1 is a rate-limiting enzyme for tryptophan metabolism, which could convert tryptophan to kynurenine and 3-hydroxyanthranilic acid [121]. Increased IDO1 expression and decreased tryptophan lead to dysfunctional effector T cells and cancer immune evasion [122]. It was reported that downregulated miR-218 protected cervical cancer cells from immune attack via elevating IDO1 level [123]. Besides, Lou et al. found that miR-448 acted as a tumor suppressive factor by targeting downstream IDO1 in colon cancer cells. The results of in vitro study showed that the ectopic expression of miR-448 was beneficial to giving full play to the functions of TILs [124]. Moreover, Huang et al. reported that miR-153 level was a core factor determining the efficacy of chimeric antigen receptor (CAR) T cells treatment in colon cancer models. In colon cancer, miR-153 directly targeted IDO1, enhancing cytotoxic activity of CAR T cells and inhibiting tumor growth [46].

### Cancer cell-derived miRNAs regulating immune evasion via exosomes or vehicles

**Fig. 3:**
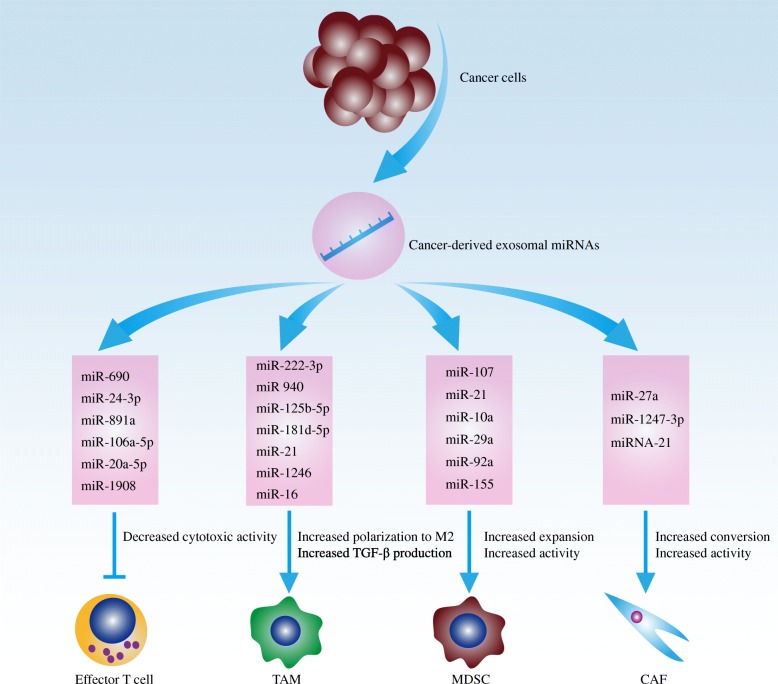
Cancer cells-derived miRNAs regulating immune evasion via exosomes or vehicles. Cancer-derived miRNAs could be packed into exosomes or microvesicles, which are transferred to numerous TILs and shape an immunosuppressive microenvironment. Cancer-derived exosomal miRNAs impair the cytotoxicity of effector cells, inducing the polarization of macrophages toward M2-like phenotype, promoting the expansion and the immunosuppressive activity of MDSCs, as well as inhibiting infiltration of lymphocytes via CAF-mediated matrix remodeling. *TIL* tumor-infiltrating lymphocyte, *MDSC* myeloid-derived suppressor cell, *CAF* cancer-associated fibroblast, *TAM* tumor-associated macrophage

Cancer-derived miRNAs not only modulate the expression profile within cancer cells but also exhibit extracellular bioactivities by exosomes or microvesicles (Table 4) [24]. Cancer-derived miRNAs could be packed into exosomes or microvesicles, which are transferred to numerous TILs and shape an immunosuppressive microenvironment (Fig. 3) [141].

**Table 4:** Cancer cells-derived miRNAs regulating immune evasion via exosomes or vehicles

Immune cells regulated by cancer cells-derived exosomal miRNAs	miRNAs	Cancer cell types	Effects on anti-cancer immune response	Ref.
Effector T cells	miR-690	Melanoma	Immunosuppressive	[125]
	miR-24-3p	NPC	Immunosuppressive	[126]
	miR-891a	NPC	Immunosuppressive	[126]
	miR-106a-5p	NPC	Immunosuppressive	[126]
	miR-20a-5p	NPC	Immunosuppressive	[126]
	miR-1908	NPC	Immunosuppressive	[126]
TAMs	miR-222-3p	EOC	Immunosuppressive	[127]
	miR-940	EOC	Immunosuppressive	[128]
	miR-21-3p	EOC	Immunosuppressive	[129]
	miR-125b-5p	EOC	Immunosuppressive	[129]
	miR-181d-5p	EOC	Immunosuppressive	[129]
	miR-21	Head and neck cancer	Immunosuppressive	[130]
	miR-1246	Colon cancer	Immunosuppressive	[131]
	miR-16	Breast cancer	Immunostimulatory	[132]
MDSCs	miR-107	Gastric cancer	Immunosuppressive	[133]
	miR-21	OSCC	Immunosuppressive	[134]
	miR-21	Glioma	Immunosuppressive	[135]
	miR-10a	Glioma	Immunosuppressive	[135]
	miR-29a	Glioma	Immunosuppressive	[136]
	miR-92a	Glioma	Immunosuppressive	[136]
	miR-155	CLL	Immunosuppressive	[137]
CAFs	miR-27a	Gastric cancer	Immunosuppressive	[138]
	miR-1247-3p	HCC	Immunosuppressive	[139]
	miR-21	HCC	Immunosuppressive	[140]

*NPC* nasopharyngeal carcinoma, *TAM* tumor-associated macrophage, *EOC* epithelial ovarian cancer, *MDSC* myeloid-derived suppressor cell, *OSCC* oral squamous cell carcinoma, *CLL* chronic lymphocytic leukemia, *CAF* cancer-associated fibroblast, *HCC* hepatocellular carcinoma

#### Cancer-derived exosomal miRNAs and effector cells

Zhou et al. reported that the apoptosis ratio of T cells increased after treated with B16 cell-derived exosomes [125]. When the release of exosome was inhibited, the abundance of TILs elevated and tumor growth was retarded [125]. Further investigation found that the cancer-derived exosomes extraordinarily increased pro-apoptotic proteins (e.g., caspase-3/7/9) and decreased anti-apoptotic proteins (e.g., BCL-2/xL and MCL-1) in CD4^+^ T cells [125]. In silico analysis indicated that a group of cancer-derived exosomal miRNAs such as miR-690 might contribute to this mitochondrial apoptosis of T cells [125]. Besides, Ye et al. found that TW03 (nasopharyngeal carcinoma cell)-derived exosomes impaired the proliferation and differentiation of T cells [126]. Meanwhile, TW03-derived exosomes remarkably downregulated the generation of cytokines including IL-2, IFN-γ, and IL-17 [126]. It was proposed that a set of commonly overexpressed miRNAs (miR-24-3p, miR-891a, miR-106a-5p, miR-20a-5p, and miR-1908) were responsible for the undermined functions of T cells [126].

#### Cancer-derived exosomal miRNAs and TAMs

Macrophages are usually categorized into pro-inflammatory (M1) populations and anti-inflammatory (M2) populations [142]. Abundant M2-like TAMs in the tumor microenvironment promote carcinogenesis by inducing angiogenesis, suppressing anti-cancer immune response, and antagonizing cancer cell apoptosis [143]. Epithelial ovarian cancer cells induced the polarization of TMAs toward M2-like phenotype via secreting exosomal miR-222-3p and miR-940 [127, 128]. Besides, under hypoxic condition, epithelial ovarian cancer cell-secreted exosomes contained miRNAs such as miR-21-3p, miR-125b-5p, and miR-181d-5p, which enhanced the polarization of M2-like TAMs and promoted cancer growth [129]. Moreover, Hsieh et al. found that some snail-overexpressed cancer cells generated miR-21-containing exosomes during epithelial-mesenchymal transition (EMT) [130]. These exosomes were engulfed by CD14^+^ monocytes, inducing the polarization toward M2-like phenotype and suppressing the expression of M1 phenotype-associated markers [130]. Similarly, Cooks et al. observed that cancer cells harboring TP53 mutation could reprogram neighboring TAMs into pro-tumor state via secreting miR-1246-enriched exosomes [131]. After uptake of exosomal miR-1246, TAMs exhibited higher immunosuppression activity with upregulated TGF-β production [131]. Notably, it was reported that epigallocatechin gallate increased the level of miR-16 in breast cancer cells (4T1 cells) which could be further transferred into TAMs by exosomes and decreased the abundance of M2-like TAMs [132].

#### Cancer-derived exosomal miRNAs and MDSCs

Accumulating evidence has indicated that cancer-derived exosomal miRNAs are capable of regulating the abundance and function of MDSCs [144]. Ren et al. reported that gastric cancer cells enhanced the expansion and activity of MDSCs by delivering miRNA-107-enriched exosomes [133]. Similarly, in the condition of hypoxia, glioma secreted exosomal miR-29a and miR-92a which enhanced the proliferation and function of MDSCs via targeting Hbp1 as well as Prkar1a [136]. Besides, this hypoxia-induced glioma could generate exosomal miR-10a and miR-21 to propel the expansion and activation of MDSCs [135]. Additionally, it was found that oral squamous cell carcinoma-derived exosomal miR-21 activated the downstream PTEN-PD-L1 pathway in MDSCs, which further enhanced the immune tolerance in the tumor microenvironment [134]. However, this exosomal-mediated MDSCs induction could be interfered by additional vitamin D treatment. Analogously, Bruns et al. found that chronic lymphocytic leukemia-derived miR-155 induced the formation of MDSCs but this process was hampered by vitamin D treatment [137].

#### Cancer cells-derived exosomal miRNAs and cancer-associated fibroblasts

As the most abundant cells of cancer stroma, cancer-associated fibroblasts (CAFs) secret multiple cytokines and extracellular matrix components such as collagens and fibronectins, which generate a physical barrier separating lymphocytes from tumor and inhibiting the infiltration of effector cells [145]. Hyperactive CAF is an unfavorable prognostic factor for patients receiving immunotherapy [44]. Cancer-derived exosomal miRNA is a vital factor accounting for increased CAFs in the tumor microenvironment. Gastric cancer-derived exosomal miR-27a promoted the transformation from normal fibroblasts toward CAFs [138]. In addition, Fang et al. found that metastatic hepatocellular carcinoma cells produced exosomal miR-1247-3p which could target B4GALT3 and activate β1-integrin-NF-κB signaling pathway in CAFs [139]. Besides, Zhou et al. observed that hepatocellular carcinoma enhanced the activity of CAFs by exosomal miRNA-21-PTEN-PDK1/AKT pathway [140].

## The effect of miRNAs on immunotherapy

Due to the substantial influence of some cancer cell-derived miRNAs on anti-cancer immune response, it is promising to develop miRNA-based diagnostic tools and therapeutic applications. In NSCLC mouse models, it was found that miR-34a suppressed PD-L1 expression by binding to its 3′UTR region. Therapeutic injection of liposomal nanoparticles containing miR-34a mimics increased the abundance of TILs and decreased the ratio of PD-1^+^CD8^+^ T cells [146]. Theoretically, this miRNA-based treatment is synergistic with following immunotherapies.

In addition, some miRNAs are determinates of efficacy of immune checkpoint inhibitors. Zhang et al. found that circFGFR1 acted as the miRNA sponge of miR-381-3p and induced the therapeutic resistance to PD-1 blockade [147]. Besides, Nakahara et al. reported that the high levels of miR-16-5p, miR-17-5p, and miR-20a-5p were the indicators of responders of melanoma patients receiving anti-PD-1 therapy [148]. Moreover, Zheng et al. observed that miR-155 induced the apoptosis of T cells by Fas-FasL pathway and upregulated the expression of PD-L1 on lymphoma cells. The results of in vivo study showed that the miR-155 overexpressed lymphoma cells were highly sensitive to PD-L1 blockade treatment [149]. More and more evidence suggested that some specific miRNA expression patterns could predict immunotherapy resistance. It was documented that a panel of miRNAs including miR-99b, miR-100, miR-125a/b, and miR-146a/b highly related with treatment resistance to immune checkpoint blockade in melanoma patients. These miRNAs could induce the conversion of myeloid cells to MDSC and herald poor immunotherapy outcomes [150].

Apart from immune checkpoint inhibitors, it has been verified that some miRNAs affect the efficacy of other immunotherapies such as CAR T cells. In xenograft tumor of human colon cancer, miR-153 inhibited the expression of IDO and enhanced the effect of CAR T cells targeting epidermal growth factor receptor [46]. In addition, Zhang et al. found that miR-143 promoted the differentiation of central memory T cells and increased the secretion of cytokines. Further investigation indicated that miR-143 overexpression boosted the specific killing activity of HER2-CAR T cells against TE-7 cells by inhibiting glucose uptake and glycolysis [151].

## Perspective and conclusion

Alteration in miRNA expression profile plays an indispensable role in carcinogenesis. Upregulated onco-miRNAs and downregulated tumor-suppressed miRNAs render cancer cells with enhanced viability and invasiveness. We noticed that more and more clinical studies try to predict the patients’ prognosis or treatment efficiency by analyzing miRNA expression profile. It was more refreshing that multiple onco-miRNA-targeted agents had been designed such as miR-RX34 liposomal. Besides, Liang et al. designed an engineered exosome which could deliver chemotherapeutic drugs 5-Fluorouracil and miR-21 inhibitor oligonucleotide to Her2^+^ colon cancer cells. This co-delivering by exosomes showed potent anti-cancer effect in mouse models and reversed the chemotherapy resistance to 5-Fluorouracil [152]. The efficacy and safety of these novel targeted therapies were undergoing evaluation. The concept of miRNA has a profound implication in understanding numerous cancer malignant biological behaviors.

Apart from participating carcinogenesis, some specific miRNA expression pattern could predict cancer immune evasion. We proposed that it would be feasible to utilize miRNA expression profiles and other parameters to construct a comprehensive framework for evaluating patients’ immune state. This evaluation is meaningful to determine further treatment options. Besides, normalizing these immunosuppressive miRNA expression patterns might have a synergistic effect with simultaneous immunotherapy.

## Data Availability

Data sharing not applicable to this article as no datasets were generated or analyzed during the current study.

## Abbreviations

3′-UTR: 3′-untranslated region
APC: Antigen presentation cells
Arg1: Arginase-1
APM: Antigen processing machinery
CAF: Cancer-associated fibroblast
CAR: Chimeric antigen receptor
CTL: Cytotoxic T lymphocyte
GPER: G-protein-coupled estrogen receptor-1
HLA: Human leukocyte antigen
IDO: Indoleamine 2, 3-dioxygenase
IFN: Interferon
IL-10: Interleukin-10
iNOS: Inducible nitric oxide synthase
LMP: Proteasome subunits latent membrane protein
MDSC: Myeloid-derived suppressor cell
MHC-I: Major histocompatibility complex class I
MICA/B: MHC-I chain-related molecules A/B
NSCLC: Non-small cell lung cancer
Ship1: Sh2 domain containing inositol phosphatase-1
SOCS-1: Suppressor of cytokine signaling 1
TAM: Tumor-associated macrophage
TGF-β: Transforming growth factor-β
TIL: Tumor-infiltrating lymphocyte
TIM-3: T cell immunoglobulin and mucin domain-containing protein 3
ULBP: UL16-binding protein
VISTA: V-domain Ig suppressor of T cell activation
TAA: Tumor-associated antigen
TAP: Transporter associated with antigen processing
TIGIT: T cell immunoreceptor with Ig and ITIM domains
